# Peyronie’s Disease: A Brief Overview

**DOI:** 10.7759/cureus.37037

**Published:** 2023-04-02

**Authors:** Oladapo Feyisetan

**Affiliations:** 1 Urology, Leighton Hospital, Crewe, GBR

**Keywords:** penile prosthesis, intralesional cch, surgical treatment, psychological impact, peyronie's disease

## Abstract

Peyronie’s disease is an acquired connective tissue disease of the tunica albuginea of the penis which usually presents with penile curvature/deformity and a palpable penile plaque. It is more common in Caucasian men over the fifth decade of life, but it is an under-reported disease. Conservative and non-surgical options are supported by limited evidence except for intralesional injection of collagenase clostridium histolyticum and have limited success. The improved outcome of surgical treatment is accompanied by the risk of erectile dysfunction. This is a brief overview of Peyronie's disease, its impact on the patient, and the available treatment options.

## Introduction and background

Peyronie's disease (PD) is an acquired connective tissue disorder of the tunica albuginea of the corpus cavernosum, characterized by islands of fibrosis and plaque formation. These may impede the uniform expansion of the cylindrical tunica albuginea during penile erection, leading to penile curvature and deformity. The cause is unknown, but the prevailing theory is one of repeated micro-trauma to the tunica during sexual intercourse followed by aberrant wound healing in susceptible individuals [1,2]. Prevalence rates of 0.4-20.3% have been published with considerable variation between countries depending on the characteristics of an interrogated cohort such as age, race, and co-morbidities. It is likely under-reported due to personal embarrassment and ignorance of the disease among the general population [3-5].

## Review

Risk factors

PD has been strongly linked with Dupuytren’s disease (DD). A study on men with DD reported the prevalence of PD-like symptoms as 26% [6]. Another found a prevalence of DD to be 22% in men with PD [7]. An inheritable form of PD was associated with the presence of DD in 78% of the affected individuals [8]. Other risk factors include Paget’s disease of the bone, Ledderhose disease, hypogonadism, diabetes, urethral manipulation and radical prostatectomy [9-11].

Clinical presentation

Patients are usually Caucasian and between 40 and 60 years of age [12,13]. The common presenting symptoms are penile curvature or deformity during erection (60-94%), often associated with penile pain (20-70%), a variable degree of erectile dysfunction (ED) and a palpable plaque. The first symptom noticed is usually penile deformity and the severity of this, the extent to which it interferes with penetrative sexual intercourse, and the degree of embarrassment are key determinants of how early patients seek medical advice. Pain occurs virtually only during erections and is present in the acute phase of PD. Pain may last up to 12 months. Nearly all patients will have a palpable plaque. The origin of the ED may be psychogenic and related to loss of body image as well as performance anxiety. It may also be vascular secondary to localized cavernous fibrosis resulting in distal flaccidity or veno-occlusive dysfunction [2,11-13].

PD is divided into acute and chronic phases. The acute phase is characterised by penile pain during erection, a soft plaque and penile curvature the severity of which may increase (21-48%), not change (36-67%) or improve (3-13%). It may last up to 12 months and is followed by the chronic phase characterized by stability in penile curvature, resolution of penile pain and a hard, calcified plaque [14-17].

Psychological impact

There is an underappreciated, significant negative impact of PD on body image, mood, sexual relationships and quality of life. Men have described themselves as “disgusting,” “ugly” and “half a man.” The imposed restrictions on sexual intercourse are linked with feelings of shame, stigmatization and social isolation, and these reinforce each other. In studies, about 80% of men with PD admit to “emotional difficulties” with up to 50% experiencing moderate (26%) to severe (21%) clinical depression as a direct result of PD [18,19]. The female partners of men with PD reported decreased sexual function, lower sexual satisfaction and decreased relationship satisfaction. There was a correlation between the degree of sexual interference caused by PD and both sexual function and satisfaction. The impact of PD on relationship satisfaction appears to correlate with the degree of negative impact on the male partner with PD. Female partners appeared far less bothered by the penile deformity than the men themselves. Female partners report an improvement in their relationship and sexual function after surgical and non-surgical treatment [20-23].

Treatment options

The goals of treatment are correction of the penile curvature or deformity, preservation of penile length and restoration of erectile function. The heterogenicity in the phase of presentation, severity and characteristics of penile deformity, quality of erectile function and patients' characteristics demand that these goals be tailored to the patient’s disease and expectations. This very variability, however, makes a comparison of data across treatment studies difficult.

Medical therapy

There is currently no high-quality evidence to support medical therapy as an effective avenue for remodelling or resorption of the penile plaque underpinning the penile deformity seen in PD [24-27].

Conservative management

The role of non-surgical management of PD, based on the current evidence, is mostly limited to the acute phase and the aims are the resolution of penile pain, inhibition of further curvature, improvement in erectile function and restoration of penile length. The current options include extracorporeal shockwave treatment (ESWT), penile traction therapy (PTT), vacuum pump devices (VP) and intralesional Collagenase Clostridium histolyticum (CCH). The evidence for these interventions derives from small studies of mixed qualities except for intra-lesional CCH which is supported by high-quality evidence.

ESWT has been shown to relieve pain but has no effect on curvature or plaque size. There is conflicting evidence for and against improvement in erectile function as assessed by International Index of Erectile Function (IIEF) scores [28,29]. When combined with phosphodiesterase type 5 (PDE-5) inhibitors, there may be an improvement in erectile function as well as the resolution of penile pain [30].

PTT has been deployed in both the acute and chronic phases of the disease and has been shown to safely reduce penile curvature by mean values of 20-30⁰. The effect on the restoration of penile length and girth is minimal [31,32].

The role of VP devices as monotherapy is unclear. One small study showed a moderate reduction in pain score and penile curvature by 5-25⁰ with the most benefit seen in patients with non-calcified plaques [33]. It does not improve erectile function.

Intralesional Therapy

Intralesional therapy using CCH is both minimally invasive and supported by the highest quality of evidence from two large, double-blind, randomised placebo-controlled studies. The principle is based on enzymatic cleavage of the plaque in conjunction with mechanical remodelling. The studies included men with stable curvatures, but no complex deformities. CCH was injected into the penile plaque 24-72 hours apart followed by penile plaque remodelling 24-72 hours later. This was done in four cycles spanning a total of 24 weeks. There was a statistically significant 17% reduction in penile curvature compared with the placebo [34,35]. A vacuum pump device can be substituted for penile remodelling [36]. The original treatment regime is both expensive and onerous [37] and even a modified regime with fewer injections and patient visits is still expensive [38]. Intralesional CCH is safe with a low complication rate. Essentially, intralesional CCH confers the most benefit to men with curvatures less than 60 degrees with no complex deformities and a good penile length and either no ED or ED highly responsive to pharmacotherapy. This is a limited subset of patients and even so, the definition of success is a moving target. Intralesional therapy using Interferon α-2b (INF α-2b) is less well supported by evidence from much smaller single-centre placebo-controlled studies. These demonstrated significant improvement in penile curvature by 25-27% as well as the reduction in pain and plaque size in men with curvature less than 60 degrees [39,40]. However, intralesional INF α-2b is associated with mild to moderate systemic side effects including myalgias, arthralgia, sinusitis, fever and flu-like symptoms. Despite its efficacy, it is not widely used [24,41].

Surgical management

Surgical correction is still the most effective and durable treatment modality for PD. It should not be performed in the acute phase and ideally only be considered once the penile curvature has been stable and painless for more than three months. The indications for surgery are deformity causing significant interference with sexual intercourse and/or inadequate penile rigidity; and patients’ desire for a more predictable outcome [11,25-27]. Surgical interventions are grouped into three broad categories.

Tunical Shortening

Plication sutures are placed into the convex surface of the tunica albuginea sometimes with incision or excision of the tunica. It was originally described by Nesbit in 1965 for the correction of congenital penile curvature [42]. It was first used to treat PD in 1979 by Pryor and Fitzpatrick [43]. Thin slices of tunica were excised prior to plication [43]. Modifications of Nesbit’s procedure and novel techniques based on similar principles (Yachia, 16-dot) have since been described with no excision of the tunica [44]. The average length loss is about 1-1.5cm and significant loss described as greater than 2cm may occur in 3-9%. This is rarely a cause of sexual dysfunction [45]. The rate of de novo ED and penile hypoesthesia is less than 10% and up to 12% respectively [11].

Tunical Lengthening

This is plaque incision (+/- limited plaque excision) and graft (PIG). The plaque on the concave surface is incised to release the tunica and the defect is covered by a graft. Plaque excision by itself has largely been abandoned. There is more pronounced mobilisation of the neurovascular bundle (NVB) and the veno-occlusive mechanism for an erection is at increased risk. The main premise for these techniques is the conservation of penile length and better correction of waist deformities. The trade-off is significantly increased risk with the rate of de novo ED up to 50% and penile hypoesthesia up to 52% [11].

Insertion of Penile Prosthesis (IPP)

This is the surgery of choice for men with PD and unresponsive ED. Insertion of an inflatable or semi-rigid, malleable penile prosthesis may be sufficient to correct the deformity but penile modelling or even PIG may be necessary as an adjunct.

The critical factors governing the type of surgery are stretched penile length, the severity of penile curvature, the presence of complex deformity, erectile function and patient preference. The choice of surgery is easier at both ends of the spectrum - Plication (+/- corporoplasty) for men with a good penile length and no complex deformities and good erectile function. IPP for men with short penile length and complex deformities and poor erectile function. In between these poles, difficult choices must be made by the patient and the surgeon with regard to the risks of post-operative penile shortening, erectile function and residual curvature.

Discussion

The 60⁰ Guide

It was canon that men with curvatures greater than 60⁰ were best served by PIG with or without IPP [26,44,46-48]. This has been challenged by favourable results from plication surgery performed for severe deformities with greater frequency than PIG [44]. The 2016 consensus panel has removed this criterion [49] and the current European Association of Urology (EAU) guideline accepts the evidence for the 60⁰ cut-off point to be insufficient. It is simply a reference guide for discussions with the patient in selecting a surgical choice.

Penile Shortening

As a rule, patient perception of penile shortening after corporal plication (CP) is often more than objective measurements and on average, the loss of length is between 1 and 1.5cm. Data from non-comparable series estimates “significant” penile shortening occurs in 3.2-8.9% of patients who undergo CP [11,45]. The alternative, plaque incision/excision and graft (PIG/PEG) has proven to be an imperfect solution for the preservation of length. There is no gold-standard graft material, and each type of graft carries a different risk for contracture with time. Moreover, surgical techniques are equally varied. While initial results are excellent in the first two years [50], shortening has been reported anywhere between 0% and 60% depending on graft material and duration of follow-up [11,47,50,51] with an associated decline in quality of an erection and patient satisfaction [51]. This means that the significantly higher risk of ED following PIG may not necessarily be balanced out by preservation of penile length in the long term.

Penile Straightening

The definition of a straight penis following reconstructive surgery has been inconsistent ranging from <10⁰ to < 20⁰ and some studies not supplying an objective measurement [51]. The 2016 expert consensus has defined as penis as functionally straight if the residual curvature of less than 20⁰ [26]. Plication, PIG/PEG and IPP are equally able to achieve penile straightening. The difference lies in the degree of manipulation of the NVB and the tunica required. Consideration should be given to how much risk is acceptable to achieve a functionally straight penis in complex deformities and if the patient will accept a reasonable reduction in curvature with reduced risk of ED as an adequate trade-off when the quest for functionally unnecessary perfection carries increased risk.

Penile Prosthesis

The two types of penile prostheses are inflatable and malleable. There appears to be no significant difference in satisfaction rates between them [26,52,53] and the decision will be agreed on by the surgeon and the patient. Implantation of a penile prosthesis is demanding and ideally, should be performed in a high-volume centre.

## Conclusions

Patients with PD are often disappointed with the dearth of medical and conservative options and those who opt for surgical correction may have unattainable expectations compounded by the enforced wait until the chronic phase of the disease. The patient satisfaction rate is high for all the surgical options but there is a drop off in 5-10 years in those who undergo PIG. A comprehensive conversation covering penile length, de novo ED, residual curvature and in the case of IPP, the risk of device failure or infection should be the norm. How much risk is acceptable in the pursuit of a cosmetically straight, but functionally unnecessary penis is a decision for the patient and surgeon to negotiate.
